# Impact of COVID‐19 Lockdown on Psychopathology at the Onset of Eating Disorders

**DOI:** 10.1002/erv.70123

**Published:** 2026-04-28

**Authors:** Elvira Anna Carbone, Cristiano Dani, Matteo Geraci, Alessia Romeo, Ana Jiménez‐Peinado, Marianna Rania, Enrico Lodovici, Valentina Zofia Cordasco, Emanuele Cassioli, Eleonora Rossi, Livio Tarchi, Valdo Ricca, Giovanni Castellini, Cristina Segura‐Garcia

**Affiliations:** ^1^ Department of Health Sciences University Magna Graecia of Catanzaro Catanzaro Italy; ^2^ Department of Health Sciences University of Florence Florence Italy; ^3^ Maimonides Biomedical Research Institute of Cordoba (IMIBIC) Córdoba Spain; ^4^ Reina Sofia University Hospital Córdoba Spain; ^5^ Department of Morphological and Sociosanitary Science University of Cordoba Córdoba Spain; ^6^ Psychiatric Unit University Hospital Renato Dulbecco Catanzaro Italy; ^7^ Department of Medical and Surgical Sciences University Magna Graecia of Catanzaro Catanzaro Italy

**Keywords:** COVID‐19, eating disorders, lockdown, onset, psychopathology

## Abstract

**Objective:**

Previous research has shown that the COVID‐19 pandemic worsened eating‐disorder (ED) symptoms, but most studies have focused on inpatients and compared only two periods: pre‐COVID and the period following the pandemic's onset. Outpatient populations, particularly those with binge eating disorder (BED) and other specified feeding or eating disorders (OSFED), have been underrepresented. It remains unclear whether the pandemic influenced the clinical presentation at ED onset. This study aimed to examine differences in general and eating‐disorder–related psychopathology at first presentation among outpatients with all ED diagnoses before, during, and after the COVID‐19 lockdown in Italy.

**Methods:**

We retrospectively reviewed clinical records and assessment data from 400 patients seeking treatment for the first time at a specialised outpatient ED service. Patients were grouped into three periods: pre‐lockdown (01.01.2019–10.06.2020), lockdown (11.06.2020–30.09.2021), and post‐lockdown (01.10.2021–31.12.2022). These periods were defined to capture the effects of the lockdown while considering the DSM‐5 requirement that symptoms persist for at least 3 months to establish a diagnosis. Multivariate analyzes of variance were used to evaluate the effects of period, diagnosis, and their interaction on sociodemographic characteristics, eating‐disorder specific symptoms, and general psychopathology.

**Results:**

Significant differences were observed across diagnoses, including BED and OSFED. During the lockdown, patients exhibited greater concerns regarding dietary restriction, binge eating, and body, weight and shape across diagnostic groups compared with pre‐lockdown. The post‐lockdown period was associated with a younger age at ED onset and earlier initiation of dieting behaviours.

**Conclusions:**

By distinguishing pre‐lockdown, lockdown, and post‐lockdown phases and accounting for the DSM‐5 diagnostic timeline, this study extends prior research beyond the conventional pre/post pandemic comparison. The COVID‐19 lockdown was linked to more severe eating‐disorder specific and anxiety‐related symptoms at illness onset in outpatients across diagnoses, while the post‐lockdown period was marked by earlier onset and increased disorder‐specific concerns.

## Highlights

- Lockdown selectively influenced state‐dependent symptoms at onset in outpatients.
- COVID‐19 lockdown triggered earlier onset and amplified eating‐related concerns.
- Core diagnostic features remained stable across pre, during, post‐lockdown periods.

## 1 Introduction and Aims

The global health emergency caused by the COVID‐19 pandemic profoundly affected mental health, including the incidence and exacerbation of eating disorders (EDs) (Taquet et al. 2021; Termorshuizen et al. 2020; Zhao et al. 2022). Evidence indicates that pandemic‐related restrictions intensified ED‐related behaviours, as individuals struggled to cope with sudden changes, social isolation, and COVID‐19–related stressors. This environment contributed both to the worsening of existing symptoms and, in some cases, to an accelerated onset of EDs (Haghshomar et al. 2022; J. Devoe et al. 2023; Monteleone et al. 2021; Nisticò et al. 2021; Sideli et al. 2021). The pandemic was associated with a 36% increase in ED symptoms and a 48% rise in hospitalizations among patients with anorexia nervosa (AN), bulimia nervosa (BN), and other food‐related disorders (J. Devoe et al. 2023). Disruptions to daily routines, such as irregular mealtimes or overstocking of food, as well as restrictions on outdoor physical activity, further exacerbated concerns about body shape and weight, increasing dysfunctional eating behaviours (Cooper et al. 2020; Coulthard et al. 2021). Psychological distress, social isolation, and limited access to care also contributed to maladaptive coping strategies, including compensatory food‐seeking behaviours (Gao et al. 2022; J. Devoe et al. 2023; Rodgers et al. 2020; Schlegl et al. 2020).

Studies have documented increases in binge eating, restrictive behaviours, purging, and heightened anxiety, depressive symptoms, and body image concerns during the pandemic (Christensen et al. 2021; Fernández‐Aranda et al. 2020; Sideli et al. 2021). Evidence further suggests that ED clinical severity and complexity worsened, particularly among inpatients, who showed higher psychopathology, greater suicidal and self‐harm behaviours, and altered psychopathological network structures compared with pre‐pandemic patients (Martini et al. 2023; Todisco et al. 2023; Longo et al. 2024; Collantoni et al. 2025).

Despite these findings, little is known about the pandemic's impact on the onset and initial clinical presentation of EDs, particularly in outpatient populations and for BED and OSFED, as most studies have focused on inpatients or predominantly AN (Munguía et al. 2025). Moreover, prior research typically compared only two periods (i.e., pre‐pandemic and post‐pandemic onset) without differentiating between pre‐lockdown, lockdown, and post‐lockdown phases. Defining these three periods is essential, as DSM‐5 criteria require symptoms to persist for at least 3 months to establish a diagnosis.

A better understanding of how the pandemic influenced the initial psychopathological profile of EDs is clinically critical, as it may inform early assessment, tailored interventions, resource allocation, and preventive strategies. The present study addresses this gap by examining whether onset characteristics and clinical features (including eating and general psychopathology) differed across pre‐lockdown, lockdown, and post‐lockdown periods among outpatients seeking treatment at specialised ED units in XXX.

## 2 Methods

We retrospectively reviewed the clinical histories and assessments of patients with EDs who sought treatment for the first time at two specialised ED outpatient university units in Catanzaro and Florence (Italy). These centres were chosen for their status as national reference units for ED care, their consistent use of standardized clinical psychometric tools, and for their complete and homogeneous data collection across the entire study period. Patients were grouped into three distinct periods reflecting the timeline of the COVID‐19 pandemic in Italy: pre‐lockdown (01.01.2019 to 10.06.2020), lockdown (11.06.2020 to 30.09.2021), and post‐lockdown (01.10.2021 to 31.12.2022). The lockdown period was defined based on official government mandates and extended by 3 months to accommodate the DSM‐5 (American Psychiatric Association 2022) minimum symptom duration required for diagnosis. Although the periods differ slightly in length, they effectively capture the pre‐pandemic baseline, the acute lockdown phase, and the early post‐lockdown recovery, providing a meaningful framework for examining pandemic‐related changes in ED presentation.

### 2.1 Participants

Anonymous data and tests results from patients with EDs at the time of their first consultation for treatment at the University Hospitals of Catanzaro and Florence (Italy) were retrieved. Inclusion criteria: individuals assigned male and female at birth; age over 14 years old; a formal ED diagnosis of AN, BN, binge eating disorder (BED) or other specified eating disorder (OSFED) according to the DSM‐5 (American Psychiatric Association 2022); the provision of a valid informed consent (i.e., patient or legal representative) for the clinical consultation. The study protocol and procedures complied with the ethical principles set out in the revised version of the Declaration of Helsinki. This retrospective study used fully anonymised data from outpatient medical records collected during routine care, with no patient involvement or identifiable information. The need for ethics approval was formally waived by the competent institutional body in accordance with local policies. Informed consent was not required. Exclusion criterion: participants with an illness duration longer than 1 year were excluded to ensure the analysis focused on features at illness onset across pandemic phases, minimising heterogeneity due to chronicity, prior treatments, and recall bias.

### 2.2 Assessment

Sociodemographic information (i.e., civil status, occupation, education), clinical characteristics (i.e., age at the time of the first visit, age of ED onset, weight, height, and body mass index (BMI)), and psychopathological variables were extracted from routine clinical evaluations. Psychopathology was assessed through standardized self‐report instruments routinely administered at intake:

- Eating Disorder Examination Questionnaire (EDE‐Q 6.0), includes 28 items that measure cognitive disorders and eating behaviours that occurred in the last 28 days (Fairburn 1994)
- Eating Disorder Inventory 2 (EDI‐2) is a self‐report measure of attitudes and behaviours related to eating, weight, and satisfaction with respect to the size and shape of body (Garner 1983; Segura‐García et al. 2015)
- Brief Symptom Inventory (BSI), the short form of the Symptom Checklist‐90‐Revised (SCL‐90‐R) to assess the presence and severity of symptoms dimensions (i.e., somatisation, obsessive‐compulsive, interpersonal sensitivity, depression, anxiety, hostility phobic anxiety, paranoid ideation, psychoticism) and the Global Severity Index (GSI) as a measure of psychological distress (Franke et al. 1983)
- Difficulties in Emotion Regulation Scale (DERS) which assesses difficulties in regulating emotions (Giromini et al. 2012)
- Barratt Impulsiveness Scale −11 (BIS‐11) to assess attentional, motor, and non‐planning impulsiveness (Fossati et al. 2001)
- Toronto Alexithymia Scale (TAS‐20) for the evaluation of alexithymia (Bagby et al. 1994).

Thus, the psychopathological assessment comprised both general (BSI, DERS, BIS‐11, TAS‐20) and ED‐related psychopathology (EDE‐Q, EDI‐2).

## 3 Data Analysis

The IBM SPSS statistics software (version 28.0) was used to create and analyse the dataset. An exploratory comparative analysis was performed between the two recruitment sites to assess potential systematic differences in all demographic and psychopathological variables; no significant or clinically relevant differences emerged after correcting for Bonferroni multiple comparisons. Based on these results, the sample was analysed as a single cohort. Descriptive analyses (i.e., frequency distribution and percentages for categorical variables and mean and standard deviation for continuous variables) were included. To address the research question regarding whether ED onset and clinical presentation differed across the three pandemic‐related periods, a multivariate analysis of variance (MANOVA) was conducted to assess the effects of the pandemic period, ED diagnostic category, and their interaction on all the psychopathological variables, adjusting for age. Tukey's post‐hoc tests were applied for significant effects. A *p*‐value < 0.05 was considered statistically significant.

## 4 Results

Because men were underrepresented in the dataset (only 9 men across both university hospitals), the statistical analyses were restricted to female patients. Of the 427 participants initially enroled, 11 were excluded due to incomplete assessments, 9 because their illness duration exceeded one year, and 7 because they had received previous treatment for EDs at other services. The final sample therefore included 400 female participants: 95 belonging to Group 1 (pre‐lockdown), 126 to Group 2 (lockdown) and 179 to Group 3 (post‐lockdown).

Tables 1 and 2 show the sociodemographic characteristics of the three groups. The pre‐lockdown, lockdown and post‐lockdown groups differed significantly in terms of diagnosis, education level, age, age at illness onset, and BMI.

**TABLE 1:** Socio‐demographics.

	Group 1 Pre‐lockdown *N* = 95		Group 2 Lockdown *N* = 126		Group 3 Post‐lockdown *N* = 179		Statistics	
	*N*	%	*N*	%	*N*	%	*χ* ^2^	*p*
Diagnosis								
*AN*	46	48	61	48	74	41	12.977	**0.043**
*BN*	33	35	34	27	49	27		
*BED*	13	14	27	21	37	21		
*OSFED*	3	3	4	3	19	11		
Civil status								
Single								
*AN*	40	87	61	100	73	99	9.231	0.510
*BN*	26	79	27	79	45	92		
*BED*	6	46	12	44	20	54		
*OSFED*	3	100	3	75	16	84		
Married								
*AN*	5	11	0	0	1	1		
*BN*	4	12	4	12	3	6		
*BED*	6	46	14	52	15	41		
*OSFED*	0	0	0	0	2	11		
Divorced								
*AN*	0	0	0	0	0	0		
*BN*	0	0	1	3	0	0		
*BED*	1	8	1	4	0	0		
*OSFED*	0	0	0	0	0	0		
N.A.								
*AN*	1	2	0	0	0	0		
*BN*	3	9	2	6	1	2		
*BED*	0	0	0	0	2	5		
*OSFED*	0	0	1	25	1	5		
Education								
Middle school I								
*AN*	18	39	21	34	40	54	16.307	**0.038**
*BN*	7	21	12	35	19	39		
*BED*	6	46	8	30	13	35		
*OSFED*	1	33	2	50	12	63		
High school II								
*AN*	16	35	26	43	29	39		
*BN*	18	55	15	44	24	49		
*BED*	4	31	14	52	16	43		
*OSFED*	2	67	2	50	5	26		
University degree								
*AN*	11	24	11	18	5	7		
*BN*	3	9	6	18	4	8		
*BED*	2	15	5	19	6	16		
*OSFED*	0	0	0	0	1	5		
N. A.								
*AN*	1	2	2	5	0	0		
*BN*	5	15	1	3	2	4		
*BED*	1	8	0	0	2	5		
*OSFED*	0	0	0	0	1	5		
Occupation								
Unpaid activity								
*AN*	1	2	0	0	0	0	20.458	0.059
*BN*	1	3	0	0	1	2		
*BED*	2	15	2	7	7	19		
*OSFED*	0	0	0	0	0	0		
Employed								
*AN*	10	22	9	15	10	14		
*BN*	9	27	10	29	8	16		
*BED*	4	31	10	37	7	19		
*OSFED*	0	0	0	0	3	16		
Unemployed								
*AN*	1	2	6	10	5	7		
*BN*	4	12	4	12	5	10		
*BED*	1	8	7	26	5	14		
*OSFED*	0	0	0	0	0	0		
Student								
*AN*	30	65	38	62	57	77		
*BN*	16	49	18	53	32	65		
*BED*	3	23	7	26	16	43		
*OSFED*	2	67	2	50	15	79		
On pension								
*AN*	0	0	0	0	0	0		
*BN*	0	0	0	0	1	2		
*BED*	1	8	0	0	0	0		
*OSFED*	0	0	0	0	0	0		
N. A.								
*AN*	4	9	8	13	2	3		
*BN*	3	9	2	6	2	4		
*BED*	2	15	0	0	2	5		
*OSFED*	1	33	2	50	1	5		

*Note:* Significant results are in bold characters.

Abbreviations: AN, anorexia nervosa; BED, binge eating disorder; BN, bulimia nervosa; N.A., not answer; OSFED, other specified feeding or eating disorder.

**TABLE 2:** Clinical characteristics.

	Group 1 Pre‐lockdown *N* = 55		Group 2 Lockdown *N* = 75		Group 3 Post‐lockdown *N* = 117					Statistics ANOVA
	Mean	SD	Mean	SD	Mean	SD	F	*p*	η^2^	Post‐hoc Tukey
Age										
*AN*	22.4	8.2	21.1	5.5	19.7	5.9	DX: 33.243	**<** **0.001**	0.208	BED > OSFED, AN, BN
*BN*	24.9	8.4	24.5	10.1	22.7	8.5	Covid: 1722	0.180		
*BED*	34.3	11.7	35.8	12.1	29.4	11.8	X: 0.845	0.536		
*OSFED*	17.3	2.3	19.8	5.4	19.7	6.5				
BMI										
*AN*	16.6	2.0	16.7	2.0	16.1	1.9	DX: 280,157	**<** **0.001**	0.701	BED > BN > OSFED > AN
*BN*	22.4	3.1	23.5	5.1	25.1	6.7	Covid: 0.695	0.500		
*BED*	40.2	7.3	38.9	10.4	37.1	7.8	X: 1855	0.088		
*OSFED*	25.3	2.6	20.8	2.5	21.7	3.8				
Age at onset										
*AN*	18.3	4.8	15.8	3.1	16.5	3.3	DX: 2414	0.069		
*BN*	16.5	4.0	18.4	5.9	14.0	4.1	Covid: 1588	0.207		
*BED*	21.0	7.0	12.0	6.2	27.0	12.8	X: 5022	**<** **0.001**	0.155	
*OSFED*	12.0	—	15.0	—	15.0	2.0				

*Note:* Significant results are in bold characters.

Abbreviations: AN, anorexia nervosa; BED, binge eating disorder; BMI, body mass index; BN, bulimia nervosa; OSFED, other specified feeding or eating disorder.

A series of MANOVAs were conducted to examine the effects of diagnostic category (i.e., AN, BN, BED, OSFED), COVID‐19 pandemic timeline (i.e., pre‐lockdown, lockdown, post‐lockdown), and their interaction on general and eating‐related psychopathology measures indicated that diagnostic category had a consistently significant effect on the dependent variables, whereas the impact of the COVID‐19 period varied across measures, with some significant interactions observed in specific contexts (Table 3).

**TABLE 3:** MANOVA.

		F	*p*	η^2^	Observed power
EDE‐Q					
Diagnosis	Wilks' Lambda	9401	**0.000**	0.115	1000
COVID period	Wilks' Lambda	2657	**0.003**	0.036	0.965
Diagnosis * COVID period	Wilks' Lambda	1735	**0.008**	0.028	0.977
EDI‐2					
Diagnosis	Wilks' Lambda	7242	**0.000**	0.217	1000
COVID period	Wilks' Lambda	736	0.803	0.027	0.616
Diagnosis * COVID period	Wilks' Lambda	0.784	0.897	0.029	0.929
BSI					
Diagnosis	Wilks' Lambda	2346	**0.000**	0.074	1000
COVID period	Wilks' Lambda	2455	**0.000**	0.078	0.998
Diagnosis * COVID period	Wilks' Lambda	1015	0.445	0.033	0.974
DERS					
Diagnosis	Wilks' Lambda	3881	**0.000**	0.079	1000
COVID period	Wilks' Lambda	649	0.824	0.014	0.420
Diagnosis * COVID period	Wilks' Lambda	0.818	0.791	0.018	0.762
BIS					
Diagnosis	Wilks' Lambda	7800	**0.000**	0.088	1000
COVID period	Wilks' Lambda	1227	0.280	0.015	0.572
Diagnosis * COVID period	Wilks' Lambda	0.491	0.982	0.009	0.372
TAS					
Diagnosis	Lambda di Wilks	3462	**0.000**	0.043	0.993
COVID period	Lambda di Wilks	5997	**0.000**	0.072	1000
Diagnosis * COVID period	Lambda di Wilks	0.841	0.686	0.016	0.650

*Note:* Significant results are in bold characters.

Abbreviations: BIS‐11, Barratt Impulsiveness Scale; BSI, Brief Symptom Inventory; DERS, Difficulties in Emotion Regulation Scale; EDE‐Q, eating disorder examination questionnaire; EDI‐2, eating disorder inventory‐2; TAS, Toronto Alexithymia Scale.

For the EDE‐Q, results showed a significant effect of diagnosis, COVID‐19 period, and COVID‐19 period × Diagnosis interaction, indicating both diagnostic differences and some temporal modulation of eating‐related symptoms. For the EDI‐2, a strong effect of diagnosis was observed, indicating a medium to large effect. However, neither COVID‐19 period nor the interaction was significant at the multivariate level. The BSI showed significant effects of both diagnosis, and COVID‐19 period, suggesting pandemic‐related increases in global psychological distress. For the DERS, only diagnosis was significant. In the BIS‐11, diagnosis again had a significant effect, whereas the COVID‐19 period and their interaction was not significant. Finally, for the TAS, there were significant effects for both diagnosis and COVID‐19 period, while the interaction was not.

Table 4 describes differences on general and eating‐related psychopathology in depth. Overall, significant differences between diagnostic categories emerged across most subscales. Notably, patients with AN consistently reported lower levels of symptomatology compared to those with BN and BED. Patients with BN exhibited the highest scores across several domains, particularly in body dissatisfaction (EDI‐2), emotion dysregulation (DERS), impulsivity (BIS‐11) and alexithymia (TAS). Temporal effects related to the COVID‐19 period were observed in selected domains. Specifically, a significant main effect of time on eating concern (EDE‐Q) was observed, with post‐lockdown participants reporting higher levels compared to those assessed pre‐lockdown. Similarly, several subscales of the BSI (i.e., somatisation, obsessive‐compulsive, interpersonal sensitivity, anxiety, hostility, phobic anxiety, paranoid ideation and, psychoticism), showed progressive increases over time, with the post‐lockdown group reporting the highest scores and indicating an overall increase in general distress symptoms following the pandemic. Interaction effects (diagnosis × COVID‐19 period) were generally non‐significant across measures, suggesting that the observed temporal trends were largely similar across diagnostic categories.

**TABLE 4:** Differences on eating and general psychopathology.

	Group 1 Pre‐lockdown *N* = 55		Group 2 Lockdown *N* = 75		Group 3 Post‐lockdown *N* = 117		Statistics ANOVA			
	Mean	SD	Mean	SD	Mean	SD	F	*p*	η^2^	Post‐hoc Tukey
EDE‐Q restraint										
*AN*	3.2	2.2	3.7	2.0	3.7	1.7	DX:9419	**<** **0.001**	0.072	BED < AN, BN, OSFED
*BN*	3.5	1.5	3.1	1.9	4.0	1.5	Covid: 2189	0.113		
*BED*	2.6	1.6	1.5	1.2	2.6	1.7	X: 1390	0.218		
*OSFED*	4.1	2.4	3.4	2.6	3.9	1.4				
EDE‐Q eating concern										
*AN*	2.5	1.5	3.1	1.5	3.3	1.4	DX: 10,348	**<** **0.001**	0.079	AN < BED, BN
*BN*	3.7	1.3	3.9	1.6	4.1	1.3	Covid: 4836	**0.008**	0.026	1 < 3
*BED*	3.5	1.4	3.4	1.3	3.7	1.6	X: 1820	0.094		
*OSFED*	2.0	0.8	2.6	1.5	3.9	0.9				
EDE‐Q shape concern										
*AN*	3.4	1.8	4.6	1.5	4.4	1.6	DX: 9793	**<** **0.001**	0.075	AN < BED, BN, OSFED
*BN*	4.7	1.3	4.8	1.5	5.3	1.0	Covid: 1174	0.310		
*BED*	5.0	1.2	5.2	0.8	4.8	1.2	X: 1820	0.094		
*OSFED*	4.8	0.5	4.4	2.1	5.2	0.8				
EDE‐Q weight concern										
*AN*	3.0	1.6	3.8	1.7	3.6	1.6	DX: 11,467	< **0.001**	0.087	AN < BED, OSFED, BN
*BN*	4.3	1.3	4.4	1.6	4.8	1.3	Covid: 1208	0.300		
*BED*	4.2	1.2	4.6	1.1	4.3	1.4	X: 0.753	0.607		
*OSFED*	3.8	0.3	4.2	2.1	4.7	1.1				
EDE‐Q tot.										
*AN*	3.1	1.6	3.8	1.5	3.8	1.5	DX: 6157	**<** **0.001**	0.048	AN < BN
*BN*	4.1	1.1	4.0	1.4	4.6	1.0	Covid: 2062	0.129		
*BED*	3.9	1.0	3.7	0.8	3.9	1.2	X: 1177	0.318		
*OSFED*	3.7	0.2	3.6	1.9	4.4	0.8				
EDI‐2 drive for thinness										
*AN*	12.3	7.9	14.9	6.3	14.0	6.2	DX:7543	0,000	0.071	AN < BED < BN < OSFED
*BN*	17.8	7.9	16.3	5.6	17.4	4.5	Covid: 0.194	0.824		
*BED*	12.8	6.0	13.1	4.0	14.0	5.5	X: 0.726	0.629		
*OSFED*	18.0	0.0	21.0	0.0	17.8	4.1				
EDI‐2 bulimia										
*AN*	2.1	3.7	3.5	6.0	2.1	3.5	DX: 49,087	**0.000**	0.331	AN < OSFED < BED < BN
*BN*	12.5	9.0	10.4	6.7	11.3	5.4	Covid: 0.393	0.675		
*BED*	7.0	6.3	7.6	4.6	9.6	5.7	X: 1298	0.258		
*OSFED*	4.5	6.4	7.5	10.6	1.8	1.9				
EDI‐2 body dissatisfaction										
*AN*	12.0	6.9	15.7	6.5	14.4	6.0	DX: 20,393	**0.000**	0.171	AN < OSFED, BN, BED
*BN*	20.5	6.2	19.3	6.6	20.0	6.2	Covid: 0.878	0.417		
*BED*	18.9	7.1	20.0	7.3	20.6	5.4	X: 0.979	0.440		
*OSFED*	18.0	1.4	24.0	4.2	19.4	7.4				
EDI‐2 ineffectiveness										
*AN*	9.4	7.2	13.9	7.7	12.5	6.9	DX: 4027	**0.008**	0.039	BN > AN, BED
*BN*	15.6	10.4	13.4	8.1	16.7	7.8	Covid: 0.930	0.396		
*BED*	10.3	6.5	10.7	8.5	12.7	7.4	X: 1221	0.295		
*OSFED*	9.0	8.5	14.0	8.5	12.2	7.9				
EDI‐2 perfectionism										
*AN*	5.8	4.3	6.3	3.9	5.8	3.8	DX: 2683	**0,.047**	0.026	BN > BED
*BN*	7.7	6.5	5.9	4.4	7.0	4.7	Covid: 0.413	0.662		
*BED*	4.2	4.3	4.2	4.3	5.3	4.3	X: 0.583	0.743		
*OSFED*	6.0	5.7	3.5	2.1	5.3	3.3				
EDI‐2 interpersonal distrust										
*AN*	7.0	5.2	8.0	5.0	7.6	4.7	DX: 2582	0.054		—
*BN*	8.6	5.5	8.5	4.4	8.6	5.1	Covid: 0.871	0.420		
*BED*	5.2	5.5	4.9	4.6	7.5	6.0	X: 0.501	0.807		
*OSFED*	6.5	4.9	7.5	0.7	8.9	5.4				
EDI‐2 interoceptive awareness										
*AN*	11.0	7.1	13.2	8.2	11.1	7.3	DX: 8177	**0.000**	0.076	BN > AN, BED
*BN*	16.2	12.0	14.2	6.8	17.3	7.8	Covid: 0.302	0.740		
*BED*	9.0	7.7	7.6	5.9	10.8	7.7	X: 0.989	0.433		
*OSFED*	10.5	0.7	10.5	9.2	11.3	6.4				
EDI‐2 maturity fears										
*AN*	9.1	4.6	11.0	5.8	9.7	6.2	DX: 1404	0.242		—
*BN*	12.9	7.4	9.9	6.4	10.6	6.4	Covid: 0.684	0.505		
*BED*	9.8	7.2	9.6	6.7	9.0	6.4	X: 1596	0.148		
*OSFED*	4.5	6.4	6.0	7.1	12.2	5.7				
EDI‐2 asceticism										
*AN*	7.1	4.4	8.5	4.6	7.4	4.0	DX: 7353	**0.000**	0.069	AN > OSFED, BED, BN
*BN*	10.9	6.5	9.3	4.9	11.0	5.3	Covid: 0.976	0.378		
*BED*	6.7	2.0	7.5	3.4	8.5	4.7	X: 1024	0.410		
*OSFED*	3.5	0.7	8.0	2.8	7.3	4.8				
EDI‐2 impulsivity										
*AN*	6.8	5.5	7.3	6.3	6.6	5.2	DX: 10,487	**0.000**	0.096	AN > OSFED, BED, BN
*BN*	12.3	10.6	10.7	7.6	10.9	7.8	Covid: 0.761	0.468		
*BED*	7.1	9.0	4.2	4.4	6.5	6.3	X: 0.484	0.820		
*OSFED*	5.5	6.4	0.5	0.7	5.2	4.1				
EDI‐2 social insecurity										
*AN*	6.9	4.5	8.7	4.8	8.9	4.5	DX: 1880	0.133		—
*BN*	10.8	6.6	8.6	4.8	10.0	5.5	Covid: 0.803	0.449		
*BED*	8.9	4.6	7.6	4.5	9.9	6.3	X: 1358	0.231		
*OSFED*	6.0	2.8	13.5	4.9	9.8	6.4				
EDI‐2 tot.										
*AN*	89.6	41.1	110.6	45.9	100.2	35.0	DX: 12,762	< **0.001**	0.114	BN > BED, AN
*BN*	145.7	72.3	126.3	47.5	140.7	46.1	Covid: 0.453	0.636		
*BED*	99.9	50.5	97.1	38.0	114.4	42.8	X 1384	0.221		
*OSFED*	92.0	7.1	116.0	41.0	111.0	39.1				
BSI somatisation										
*AN*	1.7	1.6	3.1	2.3	4.0	2.9	DX: 10,444	**<** **0.001**	0.094	AN, BN < BED, OSFED
*BN*	2.3	2.2	4.0	3.3	4.7	2.8	Covid: 15,245	**<** **0.001**	0.092	1< 2< 3
*BED*	1.2	0.8	6.3	1.7	7.0	1.9	X: 1336	0.241		
*OSFED*	5.4	6.4	6.6	1.6	7.8	1.9				
BSI obsessive‐compulsive										
*AN*	2.0	2.1	3.5	2.7	5.1	3.3	DX: 9843	**<** **0.001**	0.089	AN, BN < BED, OSFED
*BN*	2.8	3.4	4.7	3.8	6.0	3.9	Covid: 18,584	**<** **0.001**	0.110	1< 2< 3
*BED*	1.4	0.7	7.7	1.6	8.4	2.2	X: 1336	0.241		
*OSFED*	6.0	7.8	7.5	2.6	9.4	1.6				
BSI interpersonal sensitivity										
*AN*	2.1	2.4	4.1	3.5	6.0	4.4	DX: 9433	**<** **0.001**	0.086	AN, BN < BED, OSFED
*BN*	3.1	3.1	5.4	4.8	7.1	4.9	Covid: 14,831	**<** **0.001**	0.090	1< 2< 3
*BED*	1.4	0.7	9.7	2.2	9.5	2.7	X: 1401	0.214		
*OSFED*	7.1	8.7	10.1	2.6	11.0	2.8				
BSI depression										
*AN*	1.5	0.8	2.5	0.9	2.0	1.0	DX: 2495	0.060		—
*BN*	2.1	0.9	2.3	1.0	2.6	1.4	Covid: 1211	0.299		
*BED*	1.5	0.8	2.3	0.9	2.0	0.9	X: 1283	0.265		
*OSFED*	2.4	1.6	1.9	1.1	2.4	0.9				
BSI anxiety										
*AN*	1.8	1.9	3.2	2.3	4.3	2.9	DX: 8339	**<** **0.001**	0.077	AN, BN < BED < OSFED
*BN*	2.4	1.9	4.1	3.4	5.0	3.2	Covid: 15,387	**<** **0.001**	0.093	1< 2< 3
*BED*	1.3	0.9	6.0	1.4	6.5	1.9	X: 0.951	0.459		
*OSFED*	5.1	6.2	6.9	0.6	8.1	1.7				
BSI hostility										
*AN*	2.1	3.1	4.0	4.9	6.8	6.1	DX: 7614	**<** **0.001**	0.071	AN, BN < BED < OSFED
*BN*	3.0	4.4	6.3	7.0	8.2	6.8	Covid: 15,136	**<** **0.001**	0.091	1< 2< 3
*BED*	1.1	0.8	10.7	2.8	11.2	2.6	X: 1126	0.347		
*OSFED*	7.2	9.7	11.0	1.1	14.2	4.0				
BSI phobic anxiety										
*AN*	1.5	2.7	3.7	5.0	6.0	5.5	DX: 13,002	**<** **0.001**	0.115	AN, BN < BED < OSFED
*BN*	2.2	3.4	5.6	6.5	7.1	5.8	Covid: 10,154	**<** **0.001**	0.063	1 < 2 < 3
*BED*	0.7	0.8	10.4	3.2	10.4	3.4	X: 1309	0.253		
*OSFED*	11.4	15.4	13.1	2.3	13.2	4.2				
BSI paranoid ideation										
*AN*	2.1	3.1	4.6	4.8	7.3	5.9	DX: 9971	**<** **0.001**	0.090	AN, BN < BED < OSFED
*BN*	3.3	4.2	7.0	7.0	8.8	6.6	Covid: 15,566	**<** **0.001**	0.094	1 < 2 < 3
*BED*	1.4	1.0	12.0	2.7	12.3	3.4	X: 1268	0.272		
*OSFED*	9.2	12.1	11.9	1.8	14.7	3.6				
BSI psychoticism										
*AN*	1.6	2.0	3.3	3.5	5.2	4.3	DX: 7679	**<** **0.001**	0.071	AN, BN < BED, OSFED
*BN*	2.2	2.7	5.0	5.3	6.0	4.8	Covid: 15,841	**<** **0.001**	0.095	1 < 2 < 3
*BED*	0.8	0.5	7.6	1.9	9.1	2.9	X: 1101	0.362		
*OSFED*	6.0	8.0	8.2	2.3	0.2	3.8				
BSI global severity index										
*AN*	1.2	0.7	1.7	0.7	1.3	0.6	DX: 4973	**0.002**	0.047	OSFED, BED < BN, AN
*BN*	1.6	0.7	1.6	0.7	1.5	0.7	Covid: 0.205	0.815		
*BED*	1.3	0.7	0.9	0.2	1.0	0.2	X: 1552	0.161		
*OSFED*	1.1	0.5	1.0	0.2	1.2	0.3				
DERS nonacceptance of emotional responses										
*AN*	17.6	7.5	17.6	6.7	17.2	7.6	Dx: 2142	0.095		—
*BN*	21.2	5.9	17.0	7.6	20.3	6.9	Covid: 0.493	0.611		
*BED*	14.6	6.7	18.1	5.3	18.1	7.5	X: 1569	0.155		
*OSFED*	15.0	4.2	23.0	3.6	17.6	7.0				
DERS difficulty engaging in goal‐directed behaviour										
*AN*	15.3	5.0	17.6	5.5	17.2	5.2	DX: 1443	0.230		—
*BN*	18.5	4.9	17.6	6.0	18.2	5.4	Covid: 0.855	0.426		
*BED*	15.7	6.6	17.6	4.5	17.4	6.4	X: 0.583	0.744		
*OSFED*	14.5	3.5	16.0	8.7	17.7	5.4				
DERS impulse control difficulties										
*AN*	15.6	6.1	17.6	6.9	15.2	6.8	Dx: 5294	**0.001**	0.047	BN > BED, AN
*BN*	19.5	5.9	18.7	7.8	19.7	7.4	Covid: 0.448	0.639		
*BED*	14.9	6.4	15.5	6.0	16.1	6.1	X: 0.682	0.665		
*OSFED*	15.5	7.8	20.0	6.9	16.8	6.7				
DERS Lack of emotional awareness										
*AN*	11.7	7.3	11.8	5.9	13.2	6.3	DX: 6481	**<** **0.001**	0.057	OSFED > BN, AN; AN < BED
*BN*	13.2	6.4	13.0	5.9	14.6	6.1	Covid: 1156	0.316		
*BED*	16.5	4.3	16.8	4.8	15.0	3.9	X: 0.824	0.552		
*OSFED*	12.5	6.4	18.7	2.5	19.1	4.9				
DERS limited access to emotion regulation strategies										
*AN*	23.9	8.5	27.8	8.4	24.9	7.9	DX: 1315	0.269		—
*BN*	27.7	6.4	25.5	9.8	28.4	8.7	Covid: 1150	0.318		
*BED*	22.2	10.3	25.3	7.7	26.0	9.0	X: 1352	0.234		
*OSFED*	22.0	1.4	30.3	8.0	25.4	7.9				
DERS Lack of emotional clarity										
*AN*	15.3	6.1	16.5	6.2	15.8	5.6	DX: 5346	**0.001**	0.047	BED < AN, BN
*BN*	16.7	5.1	15.8	4.8	16.3	5.6	Covid: 0.086	0.917		
*BED*	13.0	5.8	12.3	3.3	12.6	5.1	X: 0.289	0.942		
*OSFED*	16.0	2.8	15.3	4.2	14.4	5.9				
DERS tot.										
*AN*	105.0	27.9	115.9	29.2	108.7	30.0	DX: 3787	**0.011**	0.034	BN > BED
*BN*	122.0	26.0	112.6	32.4	122.3	31.0	Covid: 0.616	0.541		
*BED*	96.9	32.1	105.5	24.2	105.2	28.6	X: 1011	0.418		
*OSFED*	100.0	19.8	123.3	29.3	110.8	27.8				
BIS attentional impulsiveness										
*AN*	15.8	3.6	16.8	3.9	17.5	4.0	DX: 3104	**0.027**	0.028	AN < BN
*BN*	17.5	3.6	18.6	4.4	18.5	3.7	Covid: 2270	0.105		
*BED*	16.8	4.8	16.5	3.4	17.2	4.6	X: 0.646	0.693		
*OSFED*	14.5	3.5	21.0	7.8	18.1	4.7				
BIS motor impulsiveness										
*AN*	15.0	5.0	14.6	5.0	14.9	4.4	Dx: 17,867	**<** **0.001**	0.141	AN < OSFED, BN, BED
*BN*	17.3	7.4	18.6	5.7	17.4	6.1	Covid: 0.119	0.888		
*BED*	20.2	4.6	21.1	3.9	20.3	4.4	X: 0.220	0.970		
*OSFED*	18.5	6.4	19.0	3.0	19.1	4.0				
BIS Non‐planning impulsiveness										
*AN*	25.3	5.4	26.7	6.3	25.9	5.0	DX. 10,110	**<** **0.001**	0.085	AN < BN, BED
*BN*	28.7	6.5	30.0	6.1	27.8	6.9	Covid: 0.664	0.515		
*BED*	30.4	7.1	31.3	5.1	30.3	6.4	X: 0.282	0.945		
*OSFED*	24.0	1.4	27.3	7.5	27.6	4.6				
BIS tot.										
*AN*	59.6	9.0	62.6	11.6	61.8	9.0	Dx: 9607	**<** **0.001**	0.081	AN < BN, BED
*BN*	67.4	12.7	70.9	14.0	66.9	13.5	Covid: 0.890	0.412		
*BED*	67.4	12.6	68.9	9.6	67.8	12.3	X: 0.223	0.969		
*OSFED*	60.0	7.1	67.3	17.0	64.8	9.1				
TAS‐20 difficulty identifying feelings										
*AN*	21.0	7.9	20.9	8.8	17.1	8.3	DX: 6669	**<** **0.001**	0.060	BED < AN, BN; OSFED < BN
*BN*	23.7	7.8	20.6	6.1	19.7	6.9	Covid: 1851	0.159		
*BED*	17.3	10.4	15.5	7.5	13.3	7.3	X: 0.578	0.748		
*OSFED*	11.5	6.4	19.5	7.8	14.7	4.6				
TAS‐20 difficulty describing feelings										
*AN*	25.4	29.7	44.5	59.0	77.3	78.9	DX: 4174	**0.006**	0.038	OSFED > BED, BN, AN
*BN*	31.8	46.7	67.3	86.8	102.4	96.7	Covid: 22,307	**<** **0.001**	0.125	1 < 2< 3
*BED*	13.7	4.4	83.4	64.4	130.7	73.7	X: 1481	0.184		
*OSFED*	11.0	4.2	151.0	79.2	182.8	68.3				
TAS‐20 externally oriented thinking										
*AN*	17.7	4.6	19.1	5.8	18.3	5.5	DX: 1373	0.251		—
*BN*	19.8	5.1	19.1	5.3	19.2	4.8	Covid: 0.697	0.499		
*BED*	16.9	5.0	18.3	5.6	17.7	4.6	X: 0.359	0.904		
*OSFED*	18.0	1.4	22.5	12.0	19.0	5.3				
TAS‐20 tot.										
*AN*	57.3	15.1	60.6	15.5	58.7	13.0	DX: 4265	**0.006**	0.039	BED < AN, BN
*BN*	61.6	13.7	60.3	10.9	62.4	13.2	Covid: 2191	0.113		
*BED*	51.4	14.8	53.5	13.3	53.0	13.3	X: 0.842	0.538		
*OSFED*	40.5	9.2	62.0	14.1	62.3	13.8				

*Note:* Significant results are in bold characters.

Abbreviations: AN, anorexia nervosa; BED, binge eating disorder; BIS‐11, Barratt Impulsiveness Scale; BMI, body mass index; BN, bulimia nervosa; BSI, Brief Symptom Inventory; DERS, Difficulties in Emotion Regulation Scale; EDE‐Q, eating disorder examination questionnaire; EDI‐2, eating disorder inventory‐2; OSFED, other specified feeding or eating disorder; TAS, Toronto Alexithymia Scale.

## 5 Discussion

This study examined whether the clinical presentation at illness onset of EDs differed among patients initiating treatment before, during, and after the COVID‐19 lockdown in Italy. Consistent with existing literature, diagnostic category accounted for most of the variance in both general and eating‐related psychopathology, confirming that core symptom profiles at first presentation remain strongly diagnosis‐dependent and that cross‐diagnostic comparisons should be interpreted cautiously.

In contrast, the impact of the pandemic was more selective and domain specific. Temporal differences emerged for eating‐related concerns (EDE‐Q), selected dimensions of general distress (BSI), and alexithymia (TAS), whereas no significant effects were observed for EDI‐2, DERS, or BIS‐11. These findings suggest that acute contextual stressors such as the pandemic and lockdown primarily affect state‐sensitive psychopathological domains, including anxiety‐related symptoms, bodily concerns, and emotional awareness, while leaving more stable, trait‐like constructs relatively unchanged at illness onset.

Specifically, patients presenting in the post‐lockdown period showed higher eating concern and greater psychological distress, in line with evidence showing a worsening of ED symptomatology during and after the pandemic (Longo et al. 2024; Martini et al. 2023; Todisco et al. 2023). This period was characterised by increased dietary restriction in individuals with AN and BN, more frequent binge eating episodes in those with BED, and heightened concerns regarding food, weight, and body shape across all diagnostic groups. Differences in general psychopathology were also observed, with significant effects on several BSI subscales (i.e., somatisation, obsessive‐compulsive, interpersonal sensitivity, anxiety, hostility, phobic anxiety, paranoid ideation, psychoticism) and on alexithymia (TAS), reflecting increased cognitive‐emotional dysregulation at first clinical presentation (Martini et al. 2023). These findings suggest that pandemic‐related stress acted as an exacerbating factor, particularly for anxiety‐related and somatic symptom dimensions, amplifying vulnerability rather than altering core diagnostic structures. The observed changes in eating psychopathology are plausibly attributable to pandemic‐related restrictions, including the combined effect of prolonged social isolation, disruptions in routines, increased exposure to appearance‐focused content, and reduced access to care. Increased time spent at home may have contributed to weight fluctuations and heightened body image distress (Vavassori and Donzelli 2024). Triggering compensatory behaviours such as stricter dietary restriction in AN, and increased binge eating in BN and BED (Klatzkin et al. 2018). These findings support the hypothesis of a pandemic‐related intensification of core ED symptoms at illness onset. By contrast, the absence of significant changes in other psychopathological domains may reflect their greater temporal stability or the relatively short observation window. Anxiety‐related symptoms, instead, are known to respond rapidly to contextual stressors and may therefore represent early markers of psychological impact during large‐scale crises. Nevertheless, the observed increased in somatisation, obsessive‐compulsive, interpersonal sensitivity, anxiety, hostility, phobic anxiety, paranoid ideation, psychoticism and alexithymia indicate that some domains are sensitive to environmental stress and should be monitored in clinical settings (Collantoni et al. 2025; Longo et al. 2024; Todisco et al. 2023).

Importantly, interactions between diagnostic category and pandemic period were largely absent, indicating that these temporal effects were broadly comparable across ED diagnoses. This suggests that the pandemic influenced the intensity of symptom expression at first presentation, rather than producing diagnosis‐specific modifications.

The pandemic was also associated with a younger age at ED onset consistent with recent literature indicating a notable decrease in the average age of onset during and after the COVID‐19 pandemic (Lin et al. 2024; Reas et al. 2025; Volpe et al. 2016). The observed younger clinical presentation likely reflects a broader trend towards earlier onset of EDs that may have been accelerated during lockdown. One plausible explanation is the increased socio‐cultural pressure linked to the widespread promotion of self‐imposed dieting behaviours and idealised body images through social media, particularly during periods of isolation and reduced in‐person peer interaction (Brooks et al. 2020). These factors may have encouraged self‐directed weight‐control practices acting as triggers for disordered eating in vulnerable individuals (Rodgers et al. 2020), especially among adolescents and pre‐adolescents (Haghshomar et al. 2022; Haines et al. 2010; Stice et al. 2017). This trend is clinically concerning given the established association between earlier onset, greater severity, chronicity, and treatment resistance (Grilo and Udo 2021). As such, the observed shift towards younger onset highlights the need for early screening, prevention strategies tailored to younger populations, and increased awareness among caregivers, educators, and health professionals. These findings are consistent with recent national evidence (Collantoni et al. 2025; Longo et al. 2024; Martini et al. 2023; Todisco et al. 2023), supporting the generalisability of our results within the national context.

Overall, although diagnostic category remained the strongest determinant of clinical presentation, our results point to specific domains that appear sensitive to pandemic‐related stress emphasising the need for flexible service organisation to respond to rapid shift in clinical demand.

Several limitations should be acknowledged. First, the time periods defining the three pandemic phases were based on restriction policies in XXX and may limit cross‐national comparability. Second, the post‐lockdown period was relatively short, potentially limiting the detection of longer‐term changes. Third, treatment response was not assessed, preventing conclusions about whether pandemic‐related factors influenced treatment effectiveness. Fourth, the sample included only female patients, limiting generalisability despite reflecting disorder prevalence. Fifth, although widely used, the BSI is a generic measure and does not capture ED‐specific psychopathology. Finally, behaviours such as self‐harm, suicidal ideation, and physical hyperactivity were not systematically assessed, despite being highlighted in previous studies. Future research should include both sexes, adopt disorder‐specific instruments, systematically assess high‐risk behaviours, and incorporate longer follow‐up periods to better clarify the long‐term clinical and treatment‐related consequences of the pandemic.

## 6 Conclusions

In line with the study aim of examining whether the clinical presentation at illness onset differed across pandemic phases, the findings indicate that the COVID‐19 lockdown was associated with selective changes in symptom expression rather than fundamental alterations in eating disorder phenotypes. Core diagnostic features remained stable across the pre‐, during‐, and post‐lockdown periods, supporting the hypothesis that diagnostic category remains the primary determinant of clinical presentation at first contact. However, consistent with our secondary hypotheses, pandemic‐related differences emerged in specific domains at onset, including a younger age of presentation, increased severity of eating‐related concerns, particularly restrictive behaviours and binge eating, and greater concern with body weight, shape, and dietary habits. By contrast, most dimensions of general psychopathology did not differ significantly across periods, with the exception of anxiety‐related symptoms and difficulties in emotional awareness and description. This pattern suggests that acute environmental stressors such as lockdown primarily influence state‐dependent and stress‐sensitive psychopathological dimensions, while more stable trait‐like features remain relatively unaffected at onset. Overall, these findings support a nuanced interpretation of the pandemic's impact, indicating an amplification of symptom severity and earlier onset rather than qualitative changes in disorder structure. Causal inferences remain limited by the observational design, and longer‐term follow‐up will be necessary to determine whether these differences persist over time.

## Author Contributions

**Elvira Anna Carbone:** data curation, investigation, writing – original draft. **Cristiano Dani:** data curation, investigation, writing – review and editing. **Matteo Geraci:** data curation, investigation. **Alessia Romeo:** data curation, investigation. **Ana Jiménez‐Peinado:** writing – review and editing. **Marianna Rania:** data curation, investigation. **Enrico Lodovici:** data curation, investigation. **Valentina Zofia Cordasco:** data curation, investigation. **Emanuele Cassioli:** data curation, investigation. **Eleonora Rossi:** data curation, investigation. **Livio Tarchi:** data curation, investigation. **Valdo Ricca:** data curation, investigation, writing – review and editing. **Giovanni Castellini:** data curation, methodology, supervision, writing – review and editing. **Cristina Segura‐Garcia:** conceptualization, data curation, methodology, supervision, formal analysis, writing – review and editing.

## Ethics Statement

This retrospective study used fully anonymised data from outpatient medical records collected during routine care, with no patient involvement or identifiable information. The need for ethics approval was formally waived by the competent institutional body in accordance with local policies. Informed consent was not required.

## Conflicts of Interest

The authors declare no conflicts of interest.

## Data Availability

The data that support the findings of this study are available from the corresponding author upon reasonable request.
